# Supplementary material for: The Construction and Exploration of a Comprehensive MicroRNA Centered Regulatory Network in Foxtail Millet (Setaria italica L.)
Source: Front Plant Sci. 2022 May 6;13:848474. doi: 10.3389/fpls.2022.848474 (PMC9121102; doi:10.3389/fpls.2022.848474)
Supplement: Supplementary file 6 [file Data_Sheet_6.zip › Sit-miRN3020_Seita.1G177300.1_257_TPlot.pdf]

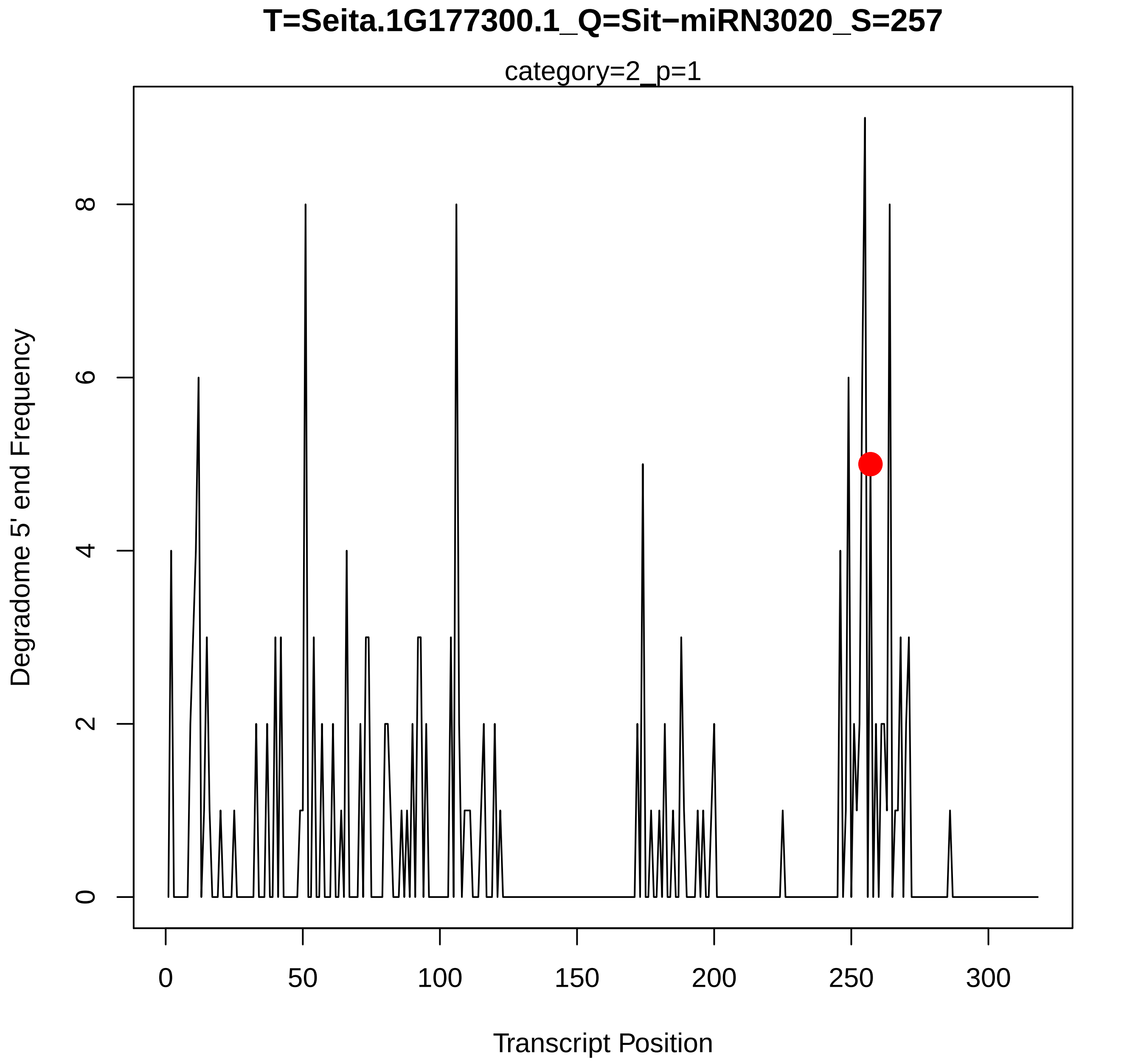
T=Seita.1G177300.1_Q=Sit−miRN3020_S=257
category=2_p=1
Degradome 5' end Frequency
0
2
4
6
8
0
50
100
150
200
250
300
Transcript Position
